# Vinorelbine – a clinical review

**DOI:** 10.1054/bjoc.2000.1203

**Published:** 2000-05-18

**Authors:** R K Gregory, I E Smith

**Affiliations:** Department of Medicine, Royal Marsden NHS Trust and Institute of Cancer Research, Fulham Road, London, SW3 6JJ, UK


*British Journal of Cancer* (2000) **82**(12), 1907–1913
© 2000 Cancer Research Campaign
doi: 10.1054/ bjoc.2000.1203, available online at http://www.idealibrary.com on IDEAL®

Review

### Vinorelbine – a clinical review

**RK Gregory and IE Smith**

Department of Medicine, Royal Marsden NHS Trust and Institute of Cancer Research, Fulham Road, London SW3 6JJ, UK

Vinorelbine is a semi-synthetic vinca-alkaloid with a broad spectrum of anti-tumour activity. The vinca-alkaloids are categorized as spindle poisons, and their mechanism of action is to interfere with the polymerization of tubulin, a protein responsible for building the microtubule system which appears during cell division.

The original vinca-alkaloids were derived from the dried leaves of the Madagascan periwinkle (*vinca rosea*), but low yields of the active compound limited the range of compounds available for study (Johnson et al, 1960). Vinblastine and vincristine were the compounds initially derived from the plant and both consisted of a cartharanthine moiety linked to a vindoline ring. Subsequently, vindesine, a desacetyl carboxyamid derivative of vinblastine, was developed.

Vinorelbine differs from the natural compounds by the presence of an eight rather than nine member catharanine ring (Figure 1). It is formulated as a light yellow amorphous powder.

### Pharmacology

Vinorelbine is a mitotic spindle poison that impairs chromosomal segregation during mitosis. It blocks cells at G2/M when present at concentrations close to IC50: at higher concentrations there is production of polyploidy (Pierre Fabre Medicament, 1993). Microtubules (derived from polymers of tubulin) are the principal target of vinorelbine (Weisenberg, 1972). The chemical modification used to produce vinorelbine allows opening of the eight-member catharanthine ring with formation of covalent reversible bond with tubulin.

The relative contribution of different microtubule-associated proteins in the production of tubulin vary between neural tissue and proliferating cells and this has important functional implications. The capacity of vinorelbine to bind preferentially to mitotic rather than axonal microtubules has been demonstrated and might imply that neurotoxicity is less likely to be a problem than with the other vinca-alkaloids (Paintrand and Pignot, 1983; Binet et al, 1989*a*). Assessment of the minimum concentration of drug required to inhibit polymerization of the spindle, compared with damage to the axonal microtubule, shows a ratio of 20:1 (Meninger et al, 1989). An additional advantage over other vinca-alkaloids lies in the selective production of mitotic tubulin paracrystallization which may point to enhanced cytotoxic action (Binet et al, 1990).

Cytotoxicity has been demonstrated against a broad spectrum of human tumour cell lines (lung, breast, leukaemia, myeloma, colon, melanoma, CNS). The $IC_{50}$ lies between 1 and 50 nMol (Ashizawa et al, 1993) (see Table 1).

*Received 23 June 1999*
*Revised 21 January 2000*
*Accepted 24 February 2000*

*Correspondence to:* IE Smith

### Molecular mechanisms of action

Like other anti-microtubule agents vinorelbine is known to be a promoter of apoptosis in cancer cells. The precise mechanisms by which this process occurs are complex and many details are yet to be elucidated. Disorganization of the microtubule structure has a number of effects, including the induction of tumour suppressor gene p53 and activation/inactivation of a number of protein kinases involved in key signalling pathways, including p21 WAF1/CIP1 and *Ras*/*Raf*, PKC/PKA (Wang et al, 1999*a*). These molecular changes result in phosphorylation and hence inactivation of the apoptosis inhibitor Bcl2 (Haldar et al, 1995). This in turn results in a decrease in the formation of hetero-dimers between Bcl2 and the pro-apoptotic gene BAX triggering the process of apoptosis in the cell (Wang et al, 1999*b*).

### Toxicology

The dose-limiting toxicity of vinorelbine is leucopenia. Transient increase in SGOT and SGPT were noted in rats and dogs but not in monkeys. In intact tectal planes from mouse embryos, vinorelbine, vincristine and vinblastine were equipotent to induce a depolymerization of mitotic microtubules, but vinorelbine was less active on axonal microtubules than the other vinca-alkaloids (Binet et al, 1989*b*).

**Table 1** Cytotoxicity of vinorelbine against human tumour cell lines

| Origin | Number of lines studied | $IC_{50}$ in nMol |
|---|---|---|
| Leukaemia | 2 | 1.59–9.38 |
| NSCLC | 8 | 1.74–19.8 |
| SCLC | 1 | 4.82 |
| Colon | 5 | 2.25–49.3 |
| Breast | 2 | 19.10 (400[a]) |
| CNS | 2 | 2.06–4.60 |
| Melanoma | 5 | 1.6–24.00 |
| Myeloma | 6 | 0.00510–0.5 |

[a] Line with MDR phenotype

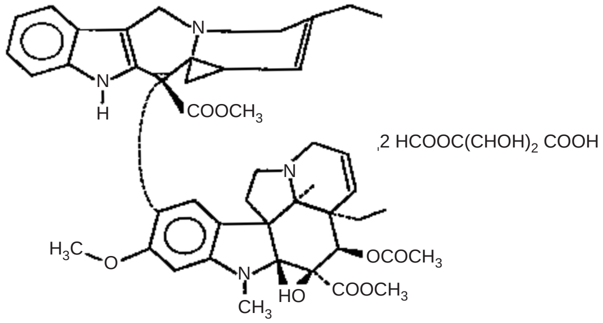

Molecular weight: 1378 (base: 778 + 2 tartric acid: 300)

**Figure 1** Chemical structure of vinorelbine

### Clinical pharmacology

The majority of data available for vinorelbine are based upon i.v. administration of the drug. Initial phase I studies of vinorelbine were performed in France, and the dose-limiting toxicity was leucopenia – at a dose of 27.5 mg $m^{-2}$ per week this was seen at grade 3 in 14% of cycles (Mathe and Reizenstein, 1985). In addition, some peripheral neuropathy was noted. Subsequent dose-finding studies, in which the dose was escalated from 30 mg $m^{-2}$ in 5 mg increments, found the MTD to be 45 mg $m^{-2}$ with the neutrophil nadir to be at day 8–10. Despite 50% of the patients sustaining grade 4 neutropenia or any grade 3 toxicity, none of the patients required hospitalization (Khayat et al, 1995). In clinical practice, many studies have demonstrated excellent dose-intensity with little significant toxicity using a schedule of 25–30 mg $m^{-2}$. i.v. Days 1 and 8 on a 21-day cycle.

### Pharmacokinetics

The pharmacokinetic properties of vinorelbine have been well defined with the development of radioimmunoassay methods (Rahmani et al, 1984) and highly sensitive high-performance liquid chromatographic (HPLC) assays using fluoresence (Debal et al, 1992), ultraviolet (Jehl et al, 1990) or electrochemical detection (Van Belle et al, 1992). The pharmacokinetic properties of intravenously administered vinorelbine can be described by a three compartment model: after a dose of 30 mg $m^{-2}$ i.v. a high initial peak of 5 umol rapidly decays to about 1 nmol at 2 h. Distribution in blood is rapid, with binding of 78% of the drug to platelets and a further 13.5% to plasma proteins with only 1.7% left as free drug in the first 2 h after administration. Subsequently, binding to plasma proteins is in the order of 70–80%. The drug diffuses freely into tissues showing a large volume of distribution and an elimination half-life of 40 h (Marquet et al, 1992).

Within 30 min of administration vinorelbine is highly concentrated in bile, excretory organs (spleen, liver and kidney), lung, muscle and heart in a range of experimental animals (Kobayashi et al, 1993). High levels of vinorelbine are found in both normal lung and tumour tissue and diffusion out of tumour tissue appears to be slow (Leveque et al, 1993). Brain and plasma levels are comparable in animal studies (Kobayashi et al, 1993). In pregnant rats drug is seen to cross the placenta and is detectable in the foetus (Van Belle et al, 1992).

There are some data available on the pharmacokinetics of the oral preparation of the drug which is currently in clinical trial. Vinorelbine has been evaluated as an oral preparation administered as a gelatin-filled capsule (Lucas et al, 1992) in women with advanced breast cancer (Spicer et al, 1994) and non-small cell lung cancer (Vokes et al, 1994). Initial studies have shown bioavailability for a soft liquid gelatin filled capsule to be 24% and for an unspecified formulation to be 40%; food did not appear to influence this (Van Cantfort et al, 1989). Within a dose range of 50–180 mg, peak plasma vinorelbine concentrations of 0.07–0.8 mg $ml^{-1}$ were seen at 1–2 h after administration of drug (Van Cartfort et al, 1989). The pharmacokinetic profile of orally administered vinorelbine is very similar to that seen with the intravenous route. The exception to this was an observed high clearance of 5.4 $h^{-1}kg^{-1}$ seen in nine patients treated in the preliminary studies, and it was proposed that this was due to first-pass hepatic metabolism (Cros et al, 1989).

### Concentration–effect relationship

Data derived from testing against cell lines indicated that the $IC_{50}$ was between 1 and 50 nmol which is a concentration range readily attained by standard dosing at 30 mg $m^{-2}$ which produces peak plasma levels of 1 μmol, with levels of 1 nmol present at 72 h (Levegue et al, 1992). Testing of the murine leukaemia model P-388 demonstrated a steep dose-response curve with response rates correlated to the total dose administered; these studies indicated that dosing at weekly intervals would be predicted to provide the most effective schedule (Cros et al, 1989).

**Table 2** Recommended dose modifications for vinorelbine in hepatic impairment

| Bilirubin | Vinorelbine dose |
|---|---|
| ≤2 mg $dl^{-1}$ | 30 mg $m^{-2}$ |
| 2.1–3 mg $dl^{-1}$ | 15 mg $m^{-2}$ |
| > 3 mg $dl^{-1}$ | 7.5 mg $m^{-2}$ |

### Metabolism

The metabolism of vinorelbine is principally hepatic; only one metabolite, deacetylvinorelbine, has been identified, the activity of which is unknown (Jehl et al, 1991). Only 11% of the drug is excreted via the renal route, the majority being eliminated through faecal excretion (Bore et al, 1989). It is not known if the drug is excreted in breast milk.

As the liver provides the main route for metabolism of the drug it may follow that patients with hepatic impairment may show increased toxicity with standard dosing but there are no available data on this. Likewise the contribution of cytochrome P450 activity to vinorelbine metabolism has potential implications in patients receiving other drugs metabolized by this route (Leveque et al, 1992). There is no evidence that glucuronidization is involved in the metabolism of vinorelbine.

### Mode of use

The drug is administered by intravenous infusion into the side-port of a running saline infusion over 6–10 min followed by a further flush through to the vein to minimize vessel irritation. Care should be taken to observe the infusion site as extravasation injuries may be severe (Rittenberg et al, 1995). In view of the potential of vinorelbine to produce painful phlebitis some centres administer it by central venous catheter only.

There are no data on patients with severe hepatic impairment, but it is recommended that the dose of vinorelbine be reduced in hepatic impairment according to the bilirubin (see Table 2). There is a lack of studies concerning renal insufficiency and administration of vinorelbine and the manufacturers advise caution before initiating therapy in this group of patients. There is no evidence that dose modifications are required in elderly patients with normal hepatic and renal function.

### CLINICAL EFFICACY

### Vinorelbine as single agent therapy in non-small cell lung cancer

So far over 15 trials with greater than 25 patients have been published using vinorelbine as a single agent in non-small cell lung cancer NSCLC and these are summarized in Table 3. The overall response rate in the published trials is 23.6%. In a recently

 *© 2000 Cancer Research Campaign*

**Table 3** Single agent vinorelbine in treatment of NSCLC

| Author | Dose (mg $m^{-2}$ per week) | Points | Overall response | Median survival (weeks) |
|---|---|---|---|---|
| Depierre et al, 1991 | 30 | 78 | 29.4% | 33 |
| Rinaldi et al, 1994 | 20 | 27 | 7% | – |
| Furuse et al, 1994 | 25 | 79 | 29.1% | 40 |
| Malzyner et al, 1991 | 30 | 36 | 33% | 29.7 |
| Crawford and O'Rourke, 1994 | 30 | 143 | 12% | 30 |
| Le Chevalier et al, 1994 | 30 | 206 | 14% | 31 |
| Gil Deza et al, 1996 | 30 | 73 | 42% | 32.5 |
| Furuse et al, 1994 | 25 | 103 | 31% | 52.4 |
| Veronesi et al, 1996 | 25–30 | 23 | 39.1% | 38.7 |
| Mattioli et al, 1997 | 30 | 15 | 20% | – |
| Gridelli et al, 1998 | 30 | 42 | 24% | 35 |
| Tononi et al, 1997 | 25 | 25 | 12% | 43 |

**Table 4** Navelbine/cisplatinum in combination in NSCLC – Phase III trials

| Author | Drug combination | Points | Response | Median survival (weeks) |
|---|---|---|---|---|
| Malzyner et al, 1991 | N 30 mg $m^{-2}$ weekly<br>Cis 100 mg $m^{-2}$ q28 | 39 | 36% | – |
| Depierre et al, 1991 | N 30 mg $m^{-2}$ weekly<br>Cis 80 mg $m^{-2}$ q21 | 116 | 48% | 33 |
| Le Chevalier et al, 1994 | N 30 mg $m^{-2}$ weekly<br>Cis 120 mg $m^{-2}$ q42 | 206 | 30% | 40 |
| Wozinak et al, 1998 | N 25 mg $m^{-2}$ weekly<br>Cis 100 mg $m^{-2}$ q28 | 206 | 26% | 34 |
| Gil Deza et al, 1996 | N 30 mg $m^{-2}$ weekly<br>Cis 100 mg $m^{-2}$ q28 | 83 | 42% | 41 |
| Gebbia et al, 1994 | N 25 mg $m^{-2}$ d1 + 8<br>Cis 100 $mg^{-2}$ q28 | 50 | 38% | – |

published phase III trial, 161 elderly patients were randomized to receive either vinorelbine or best supportive care; survival was significantly improved with vinorelbine (32% vs 14% respectively at 1 year, $P = 0.003$). In a quality of life analysis, functional scales were consistently better for vinorelbine; patients treated with vinorelbine scored better than controls for tumour-specific symptoms including pain and dyspnoea, but worse for toxicity, including constipation, nausea, hair-loss and neuropathy (The Elderly Lung Cancer Vinorelbine Italian Study, 1999). Single-agent vinorelbine seems a reasonable choice for administration in the elderly in whom polychemotherapy is not deemed possible.

### Vinorelbine in combination chemotherapy in NSCLC

Vinorelbine has also been extensively tested in combination in NSCLC, most commonly with cisplatin. The published phase III trials of cisplatin-navelbine are shown in Table 4. A multi-centre European trial randomized patients to cisplatin (120 mg $m^{-2}$ day 1 and day 29 and then 6-weekly) plus vinorelbine (30 mg $m^{-2}$ weekly) or cisplatin and vindesine (3 mg $m^{-2}$ weekly for 6 weeks and then every 2 weeks) or vinorelbine alone. The cisplatin-vinorelbine combination had a superior response-rate of 30% compared to 19% for cisplatin-vindesine and 14% for vinorelbine alone; 1-year survival rates were 35%, 27% and 30% respectively (Le Chevalier et al, 1994). A pharmaco-economic analysis based on this study concluded that the most effective regimen of vinorelbine and cisplatin added substantial benefit to vinorelbine alone or another common regimen, vindesine/cisplatin, and a cost-effectiveness within accepted limits for medical interventions (Smith et al, 1995). A subsequent randomized phase III study from the SWOG group randomized 415 patients with advanced non-small cell lung cancer to receive either cisplatin 100 mg $m^{-2}$ q28 or cisplatin 100 mg $m^{-2}$ and vinorelbine 25 mg $m^{-2}$ weekly. There was a statistically superior response-rate (12% vs 26%) and 1-year survival (20% vs 36%) in favour of the combination arm (Wozinak et al, 1998). Following this, studies have been performed comparing the cisplatin-navelbine combination with other combination regimens.

Vinorelbine has also been studied in combination with other drugs in non-small cell lung cancer. In a phase III study looking at the importance of sequencing, two-drug combinations cisplatin-vinorelbine performed well in advanced NSCLC compared to epirubicin-ifosfamide with response-rates of 47% and 21% respectively. Median overall survival was 13 months for the cisplatin-vinorelbine combination compared to 7 months for the epirubicin-ifosfamide combination ($P = 0.03$) (Colvcci et al, 1997).

Combined chemotherapy and radiotherapy is a common approach in the treatment of NSCLC. In a phase II study of 33 patients, cisplatin-vinorelbine was used as the induction regimen in patients with stage III-B disease prior to radical radiotherapy with an objective response of 48%, leading the authors to conclude that this is an effective regimen pre-radiotherapy (Felip et al, 1997).

The combination of carboplatin-vinorelbine would be an attractive alternative to cisplatin-navelbine, particularly in a more frail population, because of the lack of necessity for pre-hydration. However, one potential disadvantage of this regimen would be the possibility of severe myelosupression given that this is the dose-limiting toxicity of both agents. Two-dose escalation studies have been performed looking at this combination. In the first, patients received carboplatin at an AUC of 7 and then increasing doses of vinorelbine up to 30 mg $m^{-2}$. Patients were able to tolerate the highest dose of vinorelbine but the majority required GCSF support (Crawford et al, 1994). In a subsequent study from Italy patients received a fixed dose of vinorelbine 25 mg $m^{-2}$ in conjunc-

© 2000 Cancer Research Campaign 

**Table 5** Single agent vinorelbine in treatment of metastatic breast cancer

| Author | Dose (mg m$^{-2}$) per week | Points | Overall response | Median survival (weeks) |
|---|---|---|---|---|
| Canobbio et al, 1989 | 30 | 19 | 60% | – |
| Fumoleau et al, 1993 | 30 | 145 | 41% | 78 |
| Garcia-Conde et al, 1994 | 30 | 50 | 50% | 65 |
| Romero et al, 1994 | 30 | 44 | 41% | – |
| Weber et al, 1995 | 30 | 65 | 40% | 67 |
| Bruno et al, 1995 | 30 | 63 | 44% | 50 |
| Twelves et al, 1994 | 25 | 34 | 50% | 41 |
| Terenziani et al, 1996 | 30 | 27 | 59% | 82 |

tion with carboplatin dose escalated from 300–400 mg m$^{-2}$. At top doses 19% of patients had grade III–IV neutropenia (Colleoni et al, 1996). The response-rate for this combination is encouraging, in one phase II study 77 patients with stage III and IV NSCLC received carboplatin 350 mg m$^{-2}$ D1 and navelbine 25 mg m$^{-2}$ D1 and D8 q28: response-rate was 31% with no grade IV toxicity observed (Santamaggio et al, 1996).

Paclitaxel is another relatively new drug that has attracted interest in the treatment of NSCLC. It has been combined with vinorelbine in a study of 18 patients with refractory metastatic NSCLC, and the overall response-rate was 27% with the most common side-effects being neutropenia and peripheral neuropathy (Chang et al, 1996). Several groups have investigated the combination of ifosfamide and vinorelbine. A phase I study performed by a US group found an overall response-rate of 40% with a 1-year survival rate of 48%. The regimen was highly tolerable with some patients continuing for 10 cycles (Masters et al, 1998). Cisplatin has also been added into this combination in a phase II study involving 76 untreated patients with advanced NSCLC with a response-rate of 52% and with neutropenia being the main toxicity necessitating the use of G-CSF in 27% of cases (Baldini et al, 1996). Similar response-rates were obtained using the triple combination of cisplatin-gemcitabine-navelbine in chemo-naive patients (Fiasci et al, 1997).

### Vinorelbine as single agent therapy in metastatic breast cancer

Vinorelbine has shown a level of single-agent activity equivalent to that of many standard agents, including doxorubicin, epirubicin and mitoxantrone (1992), with a response-rate for first-line treatment of 40–60% (Fumoleau et al, 1993; Canobbio et al, 1989; Vogel et al, 1999); published trials are detailed in Table 5. The largest phase II trial on single-agent vinorelbine in metastatic breast cancer treated 145 women with 30 mg m$^{-2}$ weekly. The overall response-rate was 41%; patients with skin, lymph node and lung metastases seemed to do better than those with liver and bone metastases. Previous anthracycline as adjuvant chemotherapy did not appear to have any influence on response (Fumdeau et al, 1993). Clinical activity in the elderly shows similar response-rates to those seen in younger patients, with no need to dose-reduce (Canobbio et al, 1989). A recent study in which vinorelbine as single-agent treatment was used as first-line therapy for metastatic disease in patients over 60 showed a response of 38% with a median duration of response of 9 months, and low toxicity (Vogel et al, 1999). A phase II study of 183 patients comparing weekly vinorelbine to 3-weekly melphalan showed an improved time to progression (12 vs 8 months), an improved time to treatment failure (12 vs 8 months) and improved overall survival (35.7% vs 21.7%) with vinorelbine (Jones et al, 1995). Economic assessments of anti-cancer treatments are becoming increasingly important to allow for targetting of resources. A recent cost–benefit analysis of single agents in anthracycline pre-treated patients with metastatic breast cancer compared vinorelbine, docetaxel and paclitaxel. The authors concluded that vinorelbine provided equivalent quality-adjusted disease-free survival with economic advantage over the taxanes (Leung et al, 1999).

### Vinorelbine in combination therapy for metastatic breast cancer

Vinorelbine has principally been studied in combination with anthracyclines. Published trials are summarized in Table 6. The largest phase II study of vinorelbine/doxorubicin as first-line therapy for metastatic disease was performed in the UK with 117 patients receiving both drugs at a dose of 25 mg m$^{-2}$ on days 1 and 8 of a 21-day cycle; overall response-rate was 74% (Carmichael et al, 1997). In a randomized phase III study in 97 patients with hormonal resistant advanced breast cancer, the vinorelbine/doxorubicin combination achieved equivalent response-rates (34%) to the FAC (5-FU, adriamycin, cyclophosphamide) regimen (35%), and median duration of response was equivalent in the two groups (Blajman et al, 1996). Vinorelbine has also been studied in combination with epirubicin. In the largest published phase II series, 52 patients received vinorelbine and epirubicin both at a dose of 25 mg m$^{-2}$ weekly; the overall response rate was 77% with 75% of patients alive at 2 years (Nistico et al, 1997).

Mitoxantrone has also been tested in combination with vinorelbine. In a phase III trial patients were randomized to receive vinorelbine/mitoxantrone (MV) or a standard FAC/FEC) regimen. The objective response-rates were similar (36% for MV vs 33% for FAC/FEC). For patients who had received prior adjuvant therapy those receiving the vinorelbine combination therapy had a significantly better response rate (33% vs 13%; $P = 0.025$), survival (20 vs 15 months; $P = 0.01$) and time to progression (8 vs 5 months; $P = 0.0007$) (Namer et al, 1997).

Vinorelbine has also been tested with other drugs in the treatment of advanced breast cancer. The combination of 5-flurouracil and vinorelbine in 63 patients treated in a phase II study in France achieved a response rate of 64% with a median survival of 100 weeks (Dieras et al, 1996). Pre-clinical data suggesting synergy between vinorelbine and the taxanes have been confirmed in a phase II study of a combination of vinorelbine/paclitaxel showing a response-rate of 60% (Romerso et al, 1998). The combination of vinorelbine and paclitaxel appears particularly active. In a single-institution study from the USA, 32 patients with anthracycline pre-

 © 2000 Cancer Research Campaign

**Table 6** Navelbine/anthracycline combinations in metastatic breast cancer

| Author | Drug combination | Points | Overall response | Median survival (weeks) |
|---|---|---|---|---|
| Spielmann et al, 1994 | N 25 mg $m^{-2}$ d1+8<br>Dox 50 mg $m^{-2}$ q21 | 87 | 74% | 118 |
| Hochster et al, 1994 | N 25 mg $m^{-2}$ d1+8<br>Dox 50 mg $m^{-2}$ q21 | 62 | 57% | – |
| Blajman et al, 1996 | N 25 mg $m^{-2}$ d1+8<br>Dox 50 mg $m^{-2}$ q21 | 86 | 76% | 82 |
| Vorobiof et al, 1997 | N 25 mg $m^{-2}$ d1+8<br>Dox 50 mg $m^{-2}$ q21 | 24 | 54% | – |
| Arca et al, 1998 | N 25 mg $m^{-2}$ d1+8<br>Dox 50 mg $m^{-2}$ q21 | 70 | 68% | 68 |
| Carmichael et al, 1997 | N 25 mg $m^{-2}$ d1+8<br>Dox 25 mg $m^{-2}$ d1+8 | 117 | 74% | – |
| Chadjaa et al, 1993 | N 25 mg $m^{-2}$ d1+8<br>Epi 60 mg $m^{-2}$ q21 | 30 | 60% | 36 |
| Nistico et al, 1997 | N 25 mg $m^{-2}$/week<br>Epi 25 mg $m^{-2}$ weekly | 52 | 77% | 43 |

treated metastatic breast cancer received infusional paclitaxel and weekly vinorelbine. Doses of both drugs were escalated according to liver function and all patients received scheduled GCSF support. Overall the response-rate was 50% with 22% of patients sustaining a complete response (Ellis et al, 1999).

Finally, consideration is now being given to the use of vinorelbine combinations in neoadjuvant and adjuvant chemotherapy. The French Oncology Group treated 104 patients with primary breast cancer with induction chemotherapy with navelbine/mitoxantrone. Overall response-rate was 45% with a 7% complete response. In total 64% patients were subsequently able to undergo conservative surgery (Adenis et al, 1996). Neutropaenia was the main toxicity with 83% of patients registering grade 3 neutropaenia. In summary, vinorelbine shows single-agent response-rates comparable to standard drugs in metastatic breast cancer and with low toxicity. In combination chemotherapy randomized trials containing the drug have shown equivalent or superior response-rates to standard combinations, again with less toxicity.

### Other tumours

Vinorelbine has had some assessment in other malignancies. The combination of carboplatin-vinorelbine in small cell lung cancer has shown a response-rate of 74% (Gridelli et al, 1998) and is currently being investigated in the UK as a treatment for those patients with small cell lung cancer with a poor prognosis. In platinum-resistant ovarian carcinoma one study found single-agent activity of the drug to be 30% (Gershenson et al, 1998). Preliminary studies of vinorelbine in patients with cervical carcinoma recurring after radiotherapy found an overall response rate of 21% (Morris et al, 1998). It has been assessed in a range of B-cell malignancies as palliative treatment for extensively pre-treated patients. In patients with relapsed Hodgkin's disease 50% responded to vinorelbine with a median duration of remission of 6 months (Devizzi et al, 1994). In patients with relapsed multiple myeloma 61% of patients achieved stabilization of their disease with 16% achieving an objective response (Harousseau et al, 1997).

### Toxicity

Vinorelbine is in general a well-tolerated cytotoxic agent but it does have some important toxicities.

### *Haematological*

The main haematological toxicity seen with vinorelbine is neutropaenia and this is dose-limiting. Grade 3–4 neutropenia has been observed in 14–52% of patients treated with weekly intravenous vinorelbine; it is reversible, with a usual duration of 7–14 days, and is not cumulative, however dose adjustments have been required in up to 70% of patients (Cvittovic and Izzo, 1992). Thrombocytopaenia is rare during therapy with vinorelbine.

### *Neurological*

The vinca-alkaloids are recognized as causing neurological toxicity. Neurotoxic effects of vinorelbine include decreased deep-tendon reflexes, constipation, parasthesiae, myalgia, jaw pain and paralytic ileus. Peripheral neuropathy has been reported in up to 30% of patients receiving vinorelbine but this was severe, i.e. grade III or above, in only 1% (Navelbine Product Information). This is less than the other vinca-alkaloids; for example the incidence of peripheral neuropathy with vincristine is in the order of 57% (Cvittovic and Izzo, 1992).

### *Gastro intestinal effects*

Severe nausea and vomiting are relatively infrequent with vinorelbine. Grade 3 or 4 toxicity has been reported in only 1–3% of patients (Cvittovic and Izzo, 1992; Dubos et al, 1991). Constipation and paralytic ileus have also been reported (Cvittovic and Izzo, 1992).

### Venous access pain

Grade 3 or 4 cutaneous or venous reactions, e.g. pain on injection, venous pain and thrombophlebitis, have occurred in 5–10% of patients receiving vinorelbine (Besenval et al, 1991). This can to some extent be prevented by adequate flushing through of the vein with normal saline following injection of the drug. At the Royal Marsden Hospital these problems have been minimized by using a heat pad on the distal vein. In addition, patients receive a saline flush before and after the injection which is given over 6 min (Lisa Dougherty – CNS IV Services, personal communication).

### *Alopecia*

This affects only 10% of patients treated with vinorelbine and is of minor severity. Very few patients experience total hair loss or require a wig.

*© 2000 Cancer Research Campaign*

### *Cardiovascular effects*

Acute myocardial infarction has been reported following the administration of vinorelbine (Dubos et al, 1991).

### CONCLUSION

Vinorelbine is an active new vinca-alkaloid with a different spectrum of activity from parent compounds. In particular it has high activity in breast cancer where it is one of the most active agents currently available, and it has useful activity in non-small cell lung cancer. Its relatively low incidence of side-effects makes it a useful new addition to the treatment of breast cancer and non-small cell lung cancer, and further combination studies are warranted.

### REFERENCES

Adenis A, Vanlemmmens L, Fournier C, Hecquet B and Bonneteere J (1996) Does induction chemotherapy with a mitoxantrone/vinorelbine regimen allow a breast-conservative treatment in patients with operable loco-regional breast cancer. *Breast Cancer Res and Treat* 40: 161-169

Arca R, Fernadez E and Ivulich C (1998) Navelbine (N) and Doxorubicin (A) as first line chemotherapy in metastatic breast cancer. Proc 8th Internat Congress on anti-cancer chemotherapy: Abstract P116

Ashizawa T, Miyoshi K, Asada M, Kobayashi E, Okabe M, Morimoto M, Gomi K and Hirata T (1993) Anti-tumour activity of navelbine, a new vinca-alkaloid analog. *Gan To Kagaku Ryoho* **20**: 59–66

Baldini E, Tibaldi C, Ardizzoni A, Salvati F, Antilli A, Portalone L, Barbera S, Romano F, De Marinis F, Miglioino MR, Noseda MA, Borghini U, Crippa M, Ferrara G, Raimondi M, Fioretti M, Bandera M, Pennucci MC, Galeasso G, Cacciani GC, Lepidini G, Sunseri G, Lanfranco C, Rinaldi M and Rosso R (1998) Cisplatin-vindesine-mitomycin (MVP) vs Cisplatin-vinorelbine-ifosfamide (PIN) versus carboplatin-vinorelbine (CaN): a FONICAP phase II study. *Br J Cancer* **77**: 2367–70

Besenval M, Delgado M, Demarez JP and Krikorian A (1989) Safety and tolerance of Navelbine in phase I–II clinical trials. *Semin-Oncol* **16** (2 suppl 4): 37–40

Binet S, Fellous A, Lataste H, Krikorian A, Couzinier JP and Meniniger V (1989) In situ analysis of the action of Navelbine on microtubules using immunofluoresence. *Semin Oncol* **16** (suppl 4): 5–8

Binet S, Chaineau E, Fellous A, Lataste H, Krikorian A, Couzinier JP and Meninger V (1990) Immunofluoresence study of the action of Navelbine, vincristine and vinblastine on mitotic and axonal microtubules. *Int J Cancer* **46**: 262–266

Blajman C, Balbiani L, Block J, Bianchi R, Temperly G, Chacon R, Capa A, Coppola F, Bader M and Giachella O (1996) Phase III study: Navelbine plus adriamycin versus flurouracil plus adriamycin plus cyclophosphamide in advanced breast cancer. *Ann Oncol* **76** (7 suppl 5): Abstract P112

Bore P, Rahmani R, Van Cantfort J, Focan C and Cano JP (1989) Pharmacokinetics of a new anticancer drug, navelbine in patients. *Cancer chemother pharmacol* **23**: 247–251

Bruno S, Puerto VL, Mickiewicz E, Hegg R, Texeira LC, Gaitan L, Martinez L, Fernadez O, Otero J and Kesselring J (1995) Phase II trial of weekly IV vinorelbine as a single agent in first-line advanced breast cancer chemotherapy: The Latin-American experience. *Am J Clin Oncol* **18**: 392–396

Canobbio L, Boccardo F, Pastorini G, Brenna F, Martini C, Resasco M and Santi L (1989) Phase II study of Navelbine in advanced breast cancer. *Semin Oncol* **16**: 33–36

Carmicheal J, Hegg R, Firat D, Pawlicki M, Le bras F, Delgado FM, Danel P and Johnson SAN (1997) Navelbine and fractionated dose doxorubicin improves first line advanced breast cancer. *Br J Cancer* **75** (suppl 1): Abstract P85

Chadjaa M, Izzo J and May-Levin F (1993) Preliminary data on 4’epiadriamycin (EPI)-vinorelbine (VNB): a new active combination in advanced breast cancer. *Proc Annu Meet Am Soc Clin Oncol* 12: Abstract 152

Chang AY, Boros L, Garrow GC, Asbury RF and Hui L (1996) Ifosfamide, carboplatin, etoposide and paclitaxel chemotherpay: a dose escalation study *Semin Oncol* **23** (3 suppl 6): 74–7

Colucci G, Gebbia V, Galetta D, Riccardi F, Cariello S and Gebbia N (1997) Cisplatin and Navelbine followed by Ifosfamide and Epirubicin versus the opposite sequence in advanced unresectable stage III and IV NSCLC. *Br J Cancer* **76**: 1509–1517

Cros S, Wright M, Morimoto M, Lataste JP and Krikorian A (1989) Experimental anti-tumour activity of Navelbine. *Semin Oncol* **16**: 15–20

Cvittovic E and Izzo J (1992) The current and future place of Vinorelbine in cancer therapy. *Drugs* **44** (suppl 4): 36–45

Depierre A, Lemarie E and Dabouis G (1991) A phase II study of Navelbine (vinorelbine) in the treatment of non small cell lung cancer. *Am J Clin Oncol* **14**:115–119

Debal V, Morjani H, Millot JM, Angiboust JF, Gourdier B and Manfait M (1992) Determination of vinorelbine (Navelbine) in tumour cells by high performance liquid chromatography. *J Chromatogr Biomed Appl* **581**: 93–9

Devizzi L, Santoro LA, Bonfante LV, Viviani S, Balzarini, Valagussa P and Bonadonna G (1994) Vinorelbine: An active drug for the management of patients with heavily pre-treated Hodgkin’s disease. *Ann Oncol* **5**: 817–820

Dieras V, Extra JM, Bellisant E, Espie M, Morvan F, Pierga JY, Mignot L, Tresca P and Marty M (1996) Efficacy and tolerance of vinorelbine and flurouracil combination as first line chemotherapy of advanced breast cancer. JCO **14**: 3097–3104

Dubos C, Prevost JN, Brun J and Rousselot P (1991) Infarctus myocardique et vinorelbine. *Rev Mal Resp* **8**: 299–300

Felip E, Del Campo JM, Bodi R, Vera R, Casado S and Rubio D (1997) Cisplatin and navelbine followed by radiotherapy in the treatment of stage III and IV NSCLC patients. *Am J Clin Oncol* **20**: 404–406

Frasci G, Panza N, Comella P, Nicolella GP, Natale M, Pacilio C, Gravina A, Caputi V, Botti G and Comella G (1997) Cisplatin, Gemcitabine and Navelbine in locally advanced or metastatic NSCLC - phase II study. *Ann Oncol* **8**: 1045–1048

Fumoleau P, Delgado FM, Delozier T, Monnier A, Delgado G, Kerbrat P, Garcia-Giralt E, Keiling R, Namer M, Closon MJ, Chollet P, Lecourt L and Montcuquet P (1993) Phase II trial of weekly intravenous vinorelbine in first-line advanced breast cancer chemotherapy. *JCO* **11**: 1245–52

Furuse K, Kubota K and Kawahara M (1994) A phase II study of Vinorelbine, a new derivative of vinca alkaolid, for previously untreated advanced non small cell lung cancer. *Lung Cancer* **11**: 385–391

Garcia-Conde J, Lluch A, Martin M, Casado A, Gervasio H, De Olivra C, de Pablo JL, Gorostiago J, Giron GC and Cervantes A (1994) Phase II trial of weekly IV vinorelbine in first line advanced breast cancer chemotherapy. *Annal Oncol* **5**: 854–857

Gebbia V, Caruso M, Valenza R, Testa A, Cannata G, Verderame F, Cipolla C, Curto G, Oliveri D, Chiarenza M, Latteri MA, Di Gesu G and Gebbia N (1994) Vinorelbine plus cisplatinum for the treatment of stage IIIB and IV non small cell lung cancer. *Anticancer Research* **14**: 1247–1250

Gershenson DM, Burke DW, Morris M, Bast RC, Guaspari A, Hohneker J and Wharton JT (1998) A phase I study of a daily x3 schedule of intravenous vinorelbine for refractory epithelial ovarian carcinoma. *Gynecol-Oncol* **70**: 404–9

Gil Deza E, Balbiani L, Coppola F, Blajman C, Block JF, Giachella O, Chacon R, Capo A, Zori-Comba-A, Fein L, Polera L, Matwiejuk M, Jaremtchuk A, Muro H, Reale M, Bass C, Chiesa G, Van-Koten M and Schmilovich A (1996) Phase III study of Navelbine (NVB) vs NVB plus cisplatin in non small cell lung cancer (NSCLC) Stage IIIb or IV. *Proc-Annu-Meet-AM-Soc-Clin-Oncol* **15**: A1193

Gridelli C, Perrone F, Ianniello GP, Brancaccio L, Iaffaioli RV, Curcio C, D’Aprile M, Cioffi R, Cigolari S, Rossi A, Palazzolo G, Veltri E, Pergola M, De-Placido S, Gallo C, Monfardini S and Bianco AR (1998) Carboplatin plu vinorelbine, a new well tolerated and active regimen for the treatment of extensive-stage small-cell lung cancer: a phase II study. *JCO* **16**: 1414–9

Gridelli C, Perrone F, Gallo C, De Marinis F, Ianniello G, Cigolari S, Cariello S, Di Costanzo F, D’Aprile M, Rossi A, Migliorino R, Bartoluscci R, Bianco AR, Pergola M and Monfardini S (1997) Vinorelbine is well tolerated and active in the treatment of elderly patients with advanced non-small cell lung cancer, a two-stage phase II study. *Eur J Cancer* **33**: 392–397

Haldar S, Jena N and Croce CM (1995) Inactivation of Bcl2 by phosphorylation. *Proc Natl Acad Sci USA* **92**: 4507

Harousseau JL, Maloisel F, Sotto JJ, Facon TH, Child T, Kelsey SM, Johnson S, Descheemaker B and Moore L (1997) Vinorelbine in patients with recurrent multiple myelomas: a phase II study. *Annu Meet Am Soc Clin Oncol* **16**: Abstract 37

Hochster H (1994) A multicenter Phase II study of navelbine (NVB) and doxorubicin (DOX) as first line chemotherapy of metastatic breast cancer. Proc Annu Meet Am Soc Clin Oncol **13**: Abstract 203

Jehl F, Quoix E, Leveque D, Pauli G, Breillout F, Krikorian A and Monteil H (1991) Pharmacokinetics and preliminary metabolic fate of navelbine in humans as determined by high performance liquid chromatography. *Cancer Res* **47**: 2073–6

 *© 2000 Cancer Research Campaign*

Jehl F, Debs J and Herline C (1990) Determination of Navelbine and desacetylnavelbine in biological fluids by high performance liquid chromatography. *J Chromatogr* **525**: 225–33

Johnson IS, Wright HF, Svoboda GH and Vlantis J (1960) Antitumour principles derived from vinea rosea Linn:1. Vinca leucoblastine and leurosine. *Cancer Res* **20**: 1016–1022

Khayat D, Covelli A, Variol P, Benhamouda A, Jacques C and Bugat R (1995) Phase I and pharmacologic study of intravenous (IV) vinorelbine in patients with solid tumours. *Proc Annu Meet Am Soc Clin Oncol* **14**: Abstract 1518

Kobayashi S, Sakai T, Dalrymple PD, Wood SG and Chasseaud LF (1993) Disposition of the the novel anti-cancer agent vinorelbine ditartrate following Intravenous administration on mice, rats and dogs. *Arzneimittrl-Forschung* **43**: 1367–77

Kusunoki Y, Furuse K, Yamori S, Negros S, Katagami N, Takada K, Kinuwaki E and Niitani H (1995) Randomised phase II study of vinorelbine (VRB) versus vindesine (VDS) in previously untreated non small cell lung cancer Final results. *Proc Annu Meet Am Soc Clin Oncol* **14**: A1071 1995

Le Chevalier T, Brisgand D, Doulliard J-Y, Pujal , Alberola V, Monnier A, Riviere A, Lianes P, Chomy P, Cigarola S, Gottifred M, Ruffie P, Panizo A, Gaspard MH, Ravaioli A, Besenval M, Besson F, Martinez A, Berthaud P and Tursz T (1994) Randomised study of vinorelbine and cisplatin versus vendesine and cisplatin versus vinorelbine alone in advanced non-small-cell lung cancer: results of a European Multi-centre trial including 621 patients. *JCO* **12**: 360–367

Leveque D, Jehl F, Quoix E and Breillout F (1992) Clinical pharmacokinetics of vinorelbine alone and when combined with cisplatin. *J Clin Pharmacol* **32**: 1096–98

Leveque D, Quoix E, Dumont P, Massard G, Hentz JG, Charloux A and Jehl F (1993) Pulmonary distribution of vinorelbine in patients with non small cell lung cancer. *Cancer Chemother Pharmacol* **33**: 176–178

Lucas S, Donehower R, Rowinsky E, Wargin W, Hohneker J, Hseih A and Donehower R (1992) Weekly Navelbine liquid filled soft gelatin capsules at full therapeutic doses in patients with solid tumours. *Ann Oncol* **3** (suppl 1):125

Malzyner A, Bruno S, Piris N, Agnelli A, Gampel O, Fraga J, Le Pape I, Martinez A, Delgado FM and Delgado GL (1991) Randomised phase II study of Navelbine (NVB) vs NVB + CDDP (NP) in patients with inoperable non small cell lung cancer. *Eur J Cancer* **27** (suppl 2): Abstract S1048

Mathe G and Reizenstein P (1985) Phase I pharmacological study of a new vinca-alkaloid - Navelbine. *Cancer Lett* **27**: 285–293

Mattioli R, Imperatori L and Casadei V (1997) The impact of vinorelbine in elderly (aged >70 years) with NSCLC: a preliminary report. *Eur J Cancer* **33**: Abstract 1086

Marquet P, Lachetre G, Debord J, Eichler B, Bonnaud F and Nicot G (1992) Pharmacokinetics of vinorelbine in man. *Eur J Clin Pharmacol* **42**: 545–547

Masters GA, Hoffman PL, Drinkard LC, Samuels BL, Golomb HM and Vokes EE (1998) A review of Ifosfamide and Vinorelbine in advanced NSCLC. *Semin Oncol* **25** (1 suppl 2): 8–14

Meninger V, Binet S and Fellous A (1989) etude en immunofluorescence de l'action de la Navelbine sur differents types de microtubules. *Bull Cancer* **76**: Abstract

Morris M, Brader KR, Levenback C, Burke TW, Atkinson EN, Scott WR and Gershenson DM (1998) Phase II study of vinorelbine in advanced and recurrent squamous cell carcinoma of the cervix. *JCO* **16**:1094–8

Mouridsen HT (1992) Systemic therapy of advanced breast cancer. *Drugs* **44** (suppl 4): 17–28

Namer P, Soler-Michel P and Mefti F (1997) Is the combination of FAC/FEC always the best regimen in advanced breast cancer? Utility of mitoxantrone and navelbine as an alternative in some situations. Results of a phase III randomised trial. *Breast Cancer Res Treat* **46**: Abstract 406

Nistico G, Garufi C, Pace R, Galla DPG, Barni S, Frontini L and Terzoli E (1997) Weekly epirubicin and vinorelbine plus GCSF in metastatic breast cancer: high activity with 75% survival rate at 2 years. *Proc Am Soc Clin Oncol* **16**: Abstract 647

Non-small Cell Lung Cancer Collaborative Group (1995) Chemotherapy in non-small cell lung cancer: a meta-analysis using up-dated data on individual patients from 52 randomised clinical trials. *BMJ* **311**: 899–909

Paintrand MR and Pignot I (1983) Navelbine: an ultrastructural study of its effects. *J Electron Microsc* **32**: 115–124

Pierre Fabre Medicament (1993) Internal report. PFM259 P.36 (2)

Rahmani R, Bruno R, Iliadis A, Favre R, Just S, Barbet J and Cano JP (1984) Radioimmunoassay and preliminary pharmacokinetic studies in rats of 5'noranhydrovincristine (Navelbine). *Cancer Res* **44**: 5609–5613

Rinaldi M, Della Giulia M, Venturo I, Del Medico P, Serrone L, Capomolla and Lopez M (1994) Vinorelbine as single agent in advanced non small cell lung cancer (NSCLC). *Proc Annu Meet Am Soc Clin Oncol* **13**: Abstract 1212

Rittenberg CN, Gralla RJ and Rehmeyer TA (1995) Assessing and managing venous irritation associated with vinorelbine tartrate. *Oncol Nurs Forum* **22**: 707–710

Romero A, Rabinovich MG, Vallejo CT, Perez JE, Rodriguez R, Civas MA, Machiavelli M, Lacava JA, Langhi M and Romero-Acuna L (1994) Vinorelbine as first line chemotherapy for metastatic breast carcinoma. *J Clin Oncol* **12**: 336–341

Romero Acuna R, Langhi M, Perez J, Leone B, Machiavelli M, Lacava, Vallejo C, Romero A, Cuevas M, Ortiz E, Grasso S, Amato S, Salvadori M, Rodriguez R, Barbieri M and Romera-Acuna J (1998) Vinorelbine and Paclitaxel as first-line chemotherapy in metastatic breast cancer: final results. *Proc Annu Meet Am Soc Clin Oncol* **17**: abstract 658

Santamaggio C, Tulli E, Rinaldini M, Algeri R, Righi R, Pepi F, Ghezzi P, Andrei A and Bellaza A (1996) Carboplatin and Navelbine in the treatment of advanced NSCLC: a multi-centre phase II study. *Am J Clin Oncol* **21**: 67–71

Smith TJ, Hillner BF, Neighbors DM, McSorley PA and Le Chevalier T (1995) Economic evaluation of a randomised clinical trial comparing vinorelbine, vinorelbine plus cisplatin, and vindesine plus cisplatin for non-small cell lung cancer. *JCO* 132: 2166–2173

Spicer D, McCaskill-Stevens W, Oken M, Abrams J, Hines J, Cooper B, Craig J, Bertsch L and Hoeneker J (1994) Oral Navelbine in women with previously treated advanced breast cancer: a US multi-center phase II trial. *Proc Annu Meet Am Soc Clin Oncol* **13**: A105

Spielmann M, Dorval T, Turpin F, Antoine E, Jouve M, Maylevin F, Lacombe D, Rouesse J, Pouillart P, Tursz T and Merle S (1994) Phase II Trial of Vinorelbine/Doxorubicin as first-line therapy of advanced breast cancer. *Journal of Clinical Oncology* **12**: 1764–1770

The Elderly Lung Cancer Vinorelbine Italian Study (ELVIS) Group (1999) Effects of vinorelbine on quality of life and survival of elderly patients with advanced non-small cell lung cancer. *JNCI* **91**: 66–72

Terenziani M, Demicheli R, Brambilla C, Ferrari L, Moliterni A, Zambetti M, Caraceni A, Martini C and Bonadonna G (1996) Vinorelbine: an active, non cross resistant drug in advanced breast cancer: results from a phase II study. *Breast Cancer Res Treat* **39**: 285–291

Tononi A, Panzini I, Oliverio G, Pasquini E, Gianni L, Nicolini M and Ravaioli A (1997) Vinorelbine chemotherapy in non small cell lung cancer: experience in the elderly. *J Chemother* **9**: 304–308

Twelves C, Dobbs N and Curnow A (1994) A phase II, mulitcentre, UK study of vinorelbine in advanced breast cancer. *Br J Cancer* **70**: 990–993

Van Belle S, De Smet M, Monsaert C, Geerts F, Storme GE and Massart Dl (1992) High performance liquid chromatographic determination of Navelbine in MO4 mouse fibrosarcoma cells and biological fluids. *J Chromatogr* **576**: 351–357

Van Cantfort J and Cano JP (1989) Systemic bioavailability and pharmacokinetics of orally administered navelbine, pilot study. *Invest New Drugs* **7**: 460

Veronesi A, Crivellari D, Magri MD, Cartei G, Mansutti M, Foladore S and Monfardini S (1996) Vinorelbine treatment of advanced non-small cell lung cancer with special emphasis on elderly patients. *Eur J Cancer* **32A**:1809–1811

Vogel C, O'Rourke M, Winer E, Hochster H, Chang A, Adamkiewicz B, White R and McGuirt C (1999) Vinorelbine as first-line chemotherapy for advanced breast cancer in women 60 years of age or older. *Annals of Oncology* **10**: 397–402.

Vokes EE (1994) Oral Navelbine for advanced Stage IV NSCLC. Presented at the 7th World Conference on Lung Cancer, Colorado Springs

Vorobiof D, Goedhals L, Barnardt P, Gudgeon A, Van-Der-Merwe A, Smith L, Murray E, Pretorius D, Bassompierre P, Bergonzoli E, Delgado FM, Le-Pape I, Vurilion JP and Le-Couturier S (1997) Phase II study of IV Navelbine (NVB) and Doxorubicin (DOX) in previously untreated breast cancer (ABC). *Proc-Annu- Meet-Am-Soc-Clin-Oncol* **16**: A693

Wang LG, Liu XM, Kreis W and Budman DR (1999) The effect of antimicrotubule agents on signal transduction pathways of apoptosis: a review. *Cancer Chemother Pharmacol* **44**: 355–361

Wang LG, Xiu XM and Chao DL (1999) Activation of MAP kinase during apoptosis mediated by vinorelbine in MCF-7 cells. *Proc Am Assoc Cancer Res* **40**: 13

Weber BL, Vogel CL, Jones S, Harvey H, Hutchins L, Bigley J and Hohneker J (1995) Intravenous vinorelbine as first-line and second-line therapy in advanced breast cancer. *J Clin Oncol* **13**: 2722–2730

Weisenberg RC (1972) Microtubule formation in vitro in solutions containing low calcium concentrations. Science **117**: 1104–1105

Wozinak A, Crowley J, Balcerzak S, Weiss GR, Spiridonidids CH, Baker LH, Albain KS, Kelly K, Taylor SA, Gandara DR and Livingston RB (1998) Randomised trial comparing Cisplatin with Cisplatin plus Vinorelbine in the treatment of advanced non-small cell lung cancer: A South West Oncology Group Study. *JCO* **16**: 2459–2465

*© 2000 Cancer Research Campaign*
